# An externally validated age-related model of mean follicle density in the cortex of the human ovary

**DOI:** 10.1007/s10815-015-0501-7

**Published:** 2015-06-05

**Authors:** M. McLaughlin, T. W. Kelsey, W. H. B. Wallace, R. A. Anderson, E. E. Telfer

**Affiliations:** Institute of Cell Biology and Centre for Integrative Physiology, University of Edinburgh, Edinburgh, EH8 9XD UK; School of Computer Science, University of St. Andrews, St. Andrews, KY16 9SX UK; Department of Haematology/ Oncology, Royal Hospital for Sick Children, Edinburgh, EH9 1LF UK; MRC Centre for Reproductive Health, University of Edinburgh, Hugh Robson Building, Edinburgh, EH8 9XD UK

**Keywords:** Ovary, Modelling, Ageing, Follicles

## Abstract

**Purpose:**

The ability to accurately estimate a woman’s ovarian reserve by non-invasive means is the goal of ovarian reserve prediction. It is not known whether a correlation exists between model-predicted estimates of ovarian reserve and data generated by direct histological analysis of ovarian tissue. The aim of this study was to compare mean non-growing follicle density values obtained from analysis of ovarian cortical tissue samples against ovarian volume models.

**Methods:**

Non-growing follicle density values were obtained from 13 ovarian cortical biopsies (16-37 years). A mean non-growing follicle density was calculated for each patient by counting all follicles in a given volume of biopsied ovarian cortex. These values were compared to age-matched model generated densities (adjusted to take into consideration the proportion of ovary that is cortex) and the correlation between data sets tested.

**Results:**

Non-growing density values obtained from fresh biopsied ovarian cortical samples closely matched model generated data with low mean difference, tight agreement limits and no proportional error between the observed and predicted results.

**Conclusion:**

These findings validate the use of the adjusted population and ovarian volume models, to accurately predict mean follicle density in the ovarian cortex of healthy adult women.

## Introduction

The human ovary contains a population of non-growing follicles (NGFs). This population declines after an average peak of 300,000 NGFs at 18–22 weeks gestation to fewer than 1000 NGFs at menopause [1, 2], with the decrease largely due to atresia following follicular recruitment towards maturation. Follicle density is an important parameter in studies that compare normal and polycystic ovaries [3, 4], and in studies involving fertility preservation for women at risk of ovarian insufficiency due to surgery and/or aggressive radio- and chemotherapies [5–12], where subsequent low follicle density limits the success of in vivo and in vitro approaches to fertility restoration. In this study, mean follicle density (MFD) is taken to be the average number of NGFs in each cubic millimetre of the cortex of the ovary. This is the standard definition of MFD [4], although there are studies that define MFD as NGFs per quarter cubic millimetre [13], NGFs per gram [14], and primary or preantral follicles per cubic millimetre [6]. Cryopreservation techniques have been compared by calculating MFD before and after different freezing methods [6]; the cytotoxicity of drugs has been assessed by comparing MFD before and after in vitro exposure [15].

Deriving accurate predictions of follicle numbers from indirect methods is complicated by the uneven distribution of follicles within the ovary. Although indirect biochemical and ultrasound tests correlate with individual MFD [16], the gold standard method of determination is to directly examine the ovarian tissue sample [17]. Histological examination of ovarian cortical samples allows accurate calculation of individual MFDs and therefore follicle reserve at any age as >90 % of the NGF population is located in this region [14]. Direct examination however is an expensive and inherently destructive technique, requiring surgical procedures, tissue storage and the preparation and examination of histological samples. Indirect ex vivo methods have not been shown to be accurate to more than qualitative levels (low, medium or high density), and can also be destructive, with exposure to shortwave light from the use of fluorescent probes possibly damaging ovarian cells [18], and the use of compounds such as dimethylsulphoxide, ethanol and methanol to help the uptake of biochemical markers can also decrease NGF numbers during in situ assessment [19].

Currently there are no models for MFD calculation available: previous analyses have however separately derived the total NGF population of the ovary and ovarian volume. We have therefore combined and modified these to present a simple age-related model for MFD of the normal human ovarian cortex, needing only age as an input. We show that the predictive model provides a good approximation to MFDs calculated by direct histological examination of ovarian tissue from healthy women free from ovarian pathology and not using hormonal contraception, and hence that the model can be used as safe and inexpensive proxy for MFDs for ages from mid-teens to late thirties. The model will facilitate analysis of MFD in states of disease and following interventions, e.g., in relation to cytotoxic and other chemical exposures, as the pre-exposure MFD can be reliably estimated using the model with no tissue or examination needed until the post-exposure stage.

## Materials and methods

### The predictive model

The predictive model was derived by combining the predictions of two models in the published literature (i) an age-related normative model for the NGF population in the human ovary [1], and (ii) an age-related normative model for the volume of the human ovary [20]. For each age we set P (Age) and V (Age) as the predicted NGF population and the predicted volume respectively. The predicted mean follicle density (MFD) for an entire ovary at that age is then P (Age) divided by V (Age).

To estimate the proportion of the volume of a typical healthy ovary that consists of cortical tissue, we use the standard formula for the volume of a prolate ellipsoid: V_0_ = 4/3π × *a* × *b* × *c* where *a*, *b* and *c* are the length, breadth and depth values, with the estimate that *b* = *c* = 3/5 × a approximates the usual relationship between the three values for the human ovary.

**Fig. 1:**
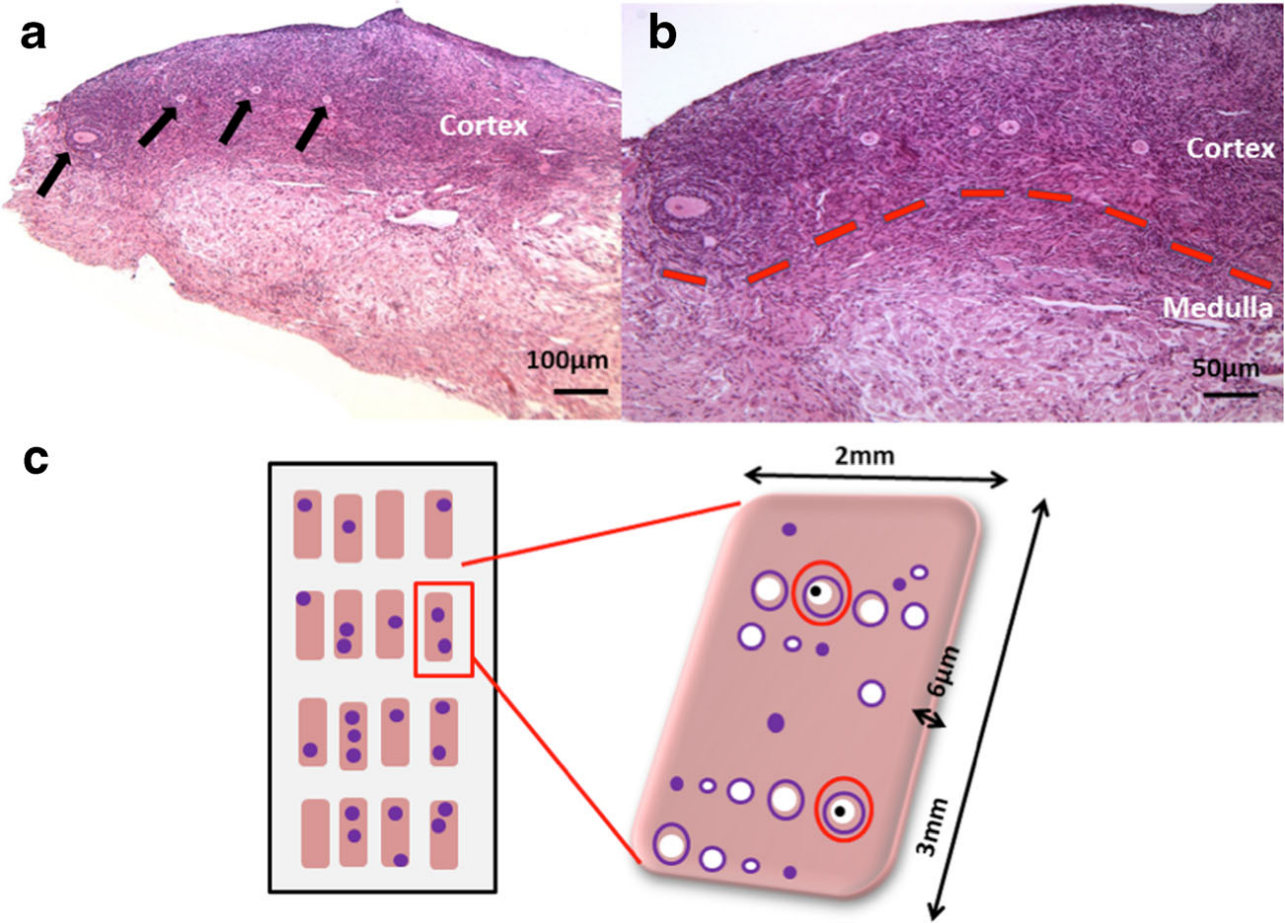
**a**. Photomicrograph of ovarian tissue from a woman aged 30 years. Growing follicles are visible in the upper cortical region (indicated by *arrows*). **b**. Follicles are present in the cortex but not in the medulla; cortical medullary interface indicated by *dotted line*. **c**. Diagrammatic representation of tissue analysis methodology. All tissue sections were examined for the presence of follicles; only sections of follicles containing the oocyte nucleolus (*black dot*) were analysed (*red circles*)

The interfaces between the epithelial, cortical and medullary regions of the ovary can be indistinct (Fig. 1a and b) however by analysis of the pixels in two-dimensional histological images of human ovarian tissue, we estimate that 3 % of the tissue area consists of the epithelial and connective layers, 10 % consists of cortex, and the remaining 87 % is the medulla. To convert these values into three dimensions, we set V_1_ = 4/3π × 0.97 × a × 3/5*a* × 3/5*a* and V_2_ = 4/3π × 0.87 × *a* × 3/5*a* × 3/5*a*. The volume of the cortex is then V_1_ – V_2_. We relate these values to chronological age by taking V_0_ to be Vol(age) – i.e., the predicted age-related volume – and re-arranging the first formula to give *a* as the cube root of 75 × Vol(age) divided by 36π. Substituting this value into V1 and V2 gives 23.8 % as the three-dimensional proportion of the ovarian volume that consists of cortical tissue, and hence the predicted MFD for a given chronological age is P(age) divided by 23.8 % of V(Age).

### Ovarian cortical biopsies

Ovarian cortical biopsies were obtained from 13 patients with informed consent and Ethical Committee approval (Table 1). All adult biopsies (*n* = 12, mean age 28.7 ± 1.4 SEM, range 22–37 years) were taken from healthy women at the time of caesarean section. The 16 year-old patient had a biopsy collected during laparoscopic ovarian cortical harvesting for fertility preservation purposes. All patients were healthy with no ovarian pathology and none were using hormonal contraception. Tissue was transferred to the laboratory in holding medium (Leibovitz medium, Life Technologies, Paisley, UK supplemented with 2 mM sodium pyruvate, 2 mM glutamine 3 mg/ml human serum albumin, 75 μg/ml penicillin G and 50 μg/ml streptomycin; all chemicals from Sigma Chemicals, Poole, UK), removed to fresh pre-warmed holding medium and examined under light microscopy. Any damaged or haemorrhagic areas were removed and the tissue was divided into fine fragments using a scalpel and fixed in neutral buffered saline for 48 h. Tissue was processed and prepared microscopic evaluation as previously described (McLaughlin et al., 2014).

**Table 2:** Predicted MFD for the age range 16–37 years

Age (years)	NGFs	Ovarian Vol. (mm^3^)	Cortical MFD
16	147,912	6358	98
17	137,487	6896	84
18	127,238	7308	73
19	117,221	7576	65
20	107,493	7700	59
21	98,106	7695	54
22	89,105	7584	49
23	80,532	7399	46
24	72,419	7169	42
25	64,794	6924	39
26	57,676	6684	36
27	51,075	6467	33
28	44,997	6283	30
29	39,438	6137	27
30	34,387	6029	24
31	29,831	5956	21
32	25,747	5911	18
33	22,112	5887	16
34	18,897	5873	14
35	16,073	5856	12
36	13,607	5825	10
37	11,468	5770	8

### Assessment of mean follicle density in ovarian biopsies

Each biopsy obtained measured between 2.5 and 8 mm^3^_._ Random samples measuring 1.2–2.2 mm in total were taken for histological analysis and each histological section of every tissue fragment was examined under light microscopy. Follicles were categorised according to their stage of development as previously described [21, 22]. To avoid over-counting, only follicle sections containing the nucleolus were assessed (Fig. 1c). The volume of tissue analysed per patient was calculated as described previously [23, 24]. Briefly, tissue volume was calculated as the sum of the area in mm^2^ of all tissue sections analyzed per patient, multiplied by 0.006 mm (the thickness of each section) to give a value in mm^3^. Mean follicle density was determined by dividing the total number of follicles per patient by the volume of tissue analyzed and expressing this value as follicles per cubic millimeter [23].

### Comparative published data on mean follicle densities

Observed MFDs were taken from Table 2 of Schmidt et al., 2003 [25] (*n* = 15, age range 13–38 years), excluding subjects who had had previous chemotherapy. These MFD values were obtained by counting follicles in every second slide, with correction factors used to account for missing follicles and follicles counted more than once. The cortical volume was estimated from two-dimensional values taken from every fourth section.

**Table 1:** Subject data for observed MFDs (*n* = 13)

Age (years)	Procedure	Tissue analysed (mm^3^)	No. NGFs counted	Observed MFD NGF/mm^3^	Predicted MFD NGF/mm^3^
16	Laparoscopy for fertility preservation	2.1	189	90.0	98
22	ECS	1.17	95	81.8	49
23	ECS	1.63	81	49.7	46
23	ECS	2.23	62	27.8	46
25	ECS	1.70	61	35.9	39
27	ECS	1.70	57	33.5	33
29	ECS	1.70	41	24.0	27
30	ECS	1.68	33	19.7	24
30	ECS	1.55	46	29.6	24
32	ECS	1.62	40	24.7	18
33	ECS	1.75	44	25.1	16
33	ECS	1.52	42	27.7	16
37	ECS	1.64	16	9.8	8

ECS denotes elective caesarean section

**Table 3:** Criteria for external validation of the predictive model by the observations, together with results obtained from correlation and Bland-Altman analysis

Criterion	Cutoff value	Own data (*n* = 13)	Published data (*n* = 15)
Correlation	*r* > 0.80	***r*** = **0.87**	***r*** = **0.90**
Mean difference	<5 NGFs	**2.6 NGFs**	**3.3 NGFs**
Limits of agreement	1 SD < 15 NGFs	**1 SD** = **11.9 NGFs**	1 SD = 20.8 NGFs
Proportional error	r^2^ < 0.10	**r** ^**2**^ = **0.003**	r^2^ = 0.12

Bold font indicates that a criterion was met or exceeded. SD denotes standard deviation of the means and differences of observations and predictions as shown in Fig. 4. Published data were taken from Table 2 of [25]

### Statistical analysis

The a priori criteria for external validation of the model by the observed data, both derived for this study and previously published [25], were (i) at least 80 % positive correlation between observed and predicted values, (ii) low average difference (5 follicles or fewer) between observed and predicted values across the entire age range, (iii) tight limits of agreement (a standard deviation of 15 follicles or fewer), and (iv) low proportional error (i.e., means and differences of observed and predicted values do not have a coefficient of determination r^2^ greater than 10 %).

Correlation between observed MFDs and predicted MFDs was measured by calculating the Pearson product-moment correlation coefficient. The mean difference between observed and predicted values, and limits of agreement at one and two standard deviations from mean were derived from Bland-Altman analysis [26, 27]. Proportional error was calculated as the correlation between the means and differences derived from the Bland-Altman plot, with absence of correlation indicating low proportional error. All analyses were performed using R version 2.15.3.

## Results

Predicted NGF populations, ovarian volumes, and cortical MFD for ages 16 through 37 are given in Table 2. Predicted cortical MFD is obtained by dividing predicted NGFs by 23.8 % of the predicted ovarian volume.

### Own data

**Fig. 2:**
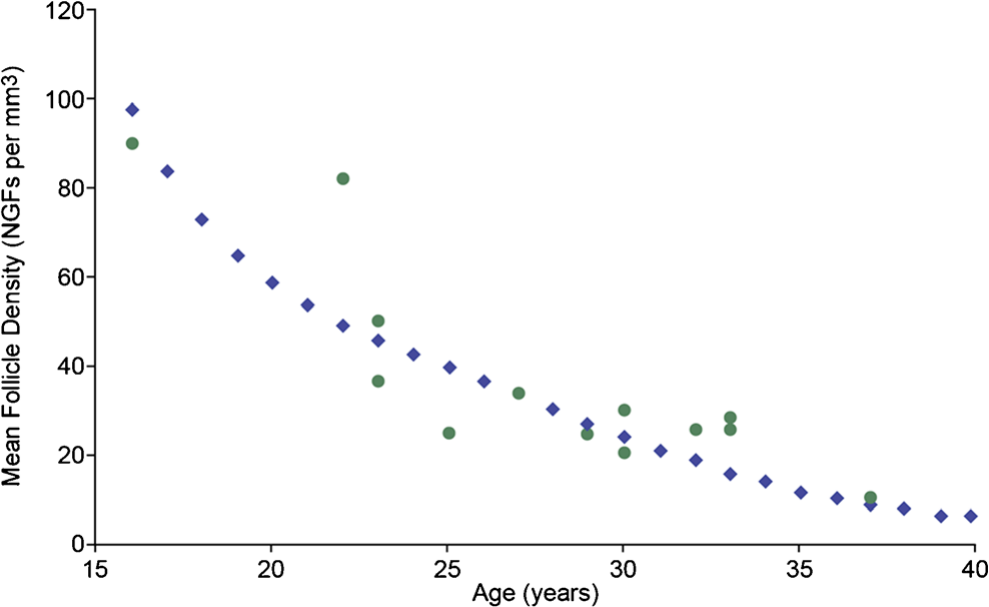
Observed and predicted MFD – predicted values are shown for ages 15 through 40 years in *blue*. Observed values (*n* = 13) are shown as *green dots*

**Fig. 3:**
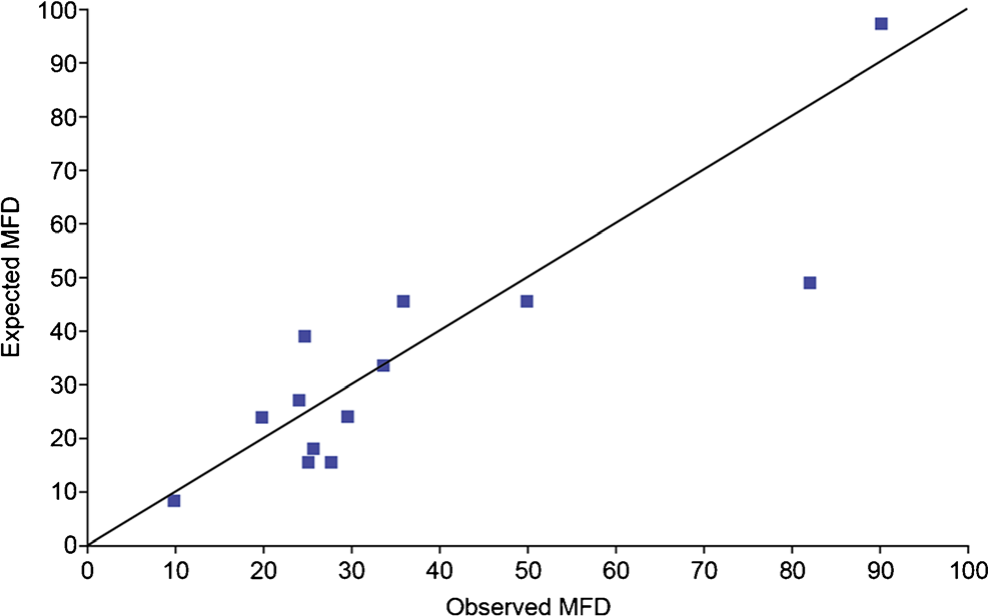
Observed vs predicted MFD - the *line* of identity represents the idealised confluence of observed and predicted values. *Blue squares* indicate observed against predicted MFD values (*n* = 13). The Pearson product-moment correlation coefficient for observed against predicted MFDs is 0.87 (95 % CI 0.62–0.96, *p* = 0.001)

**Fig. 4:**
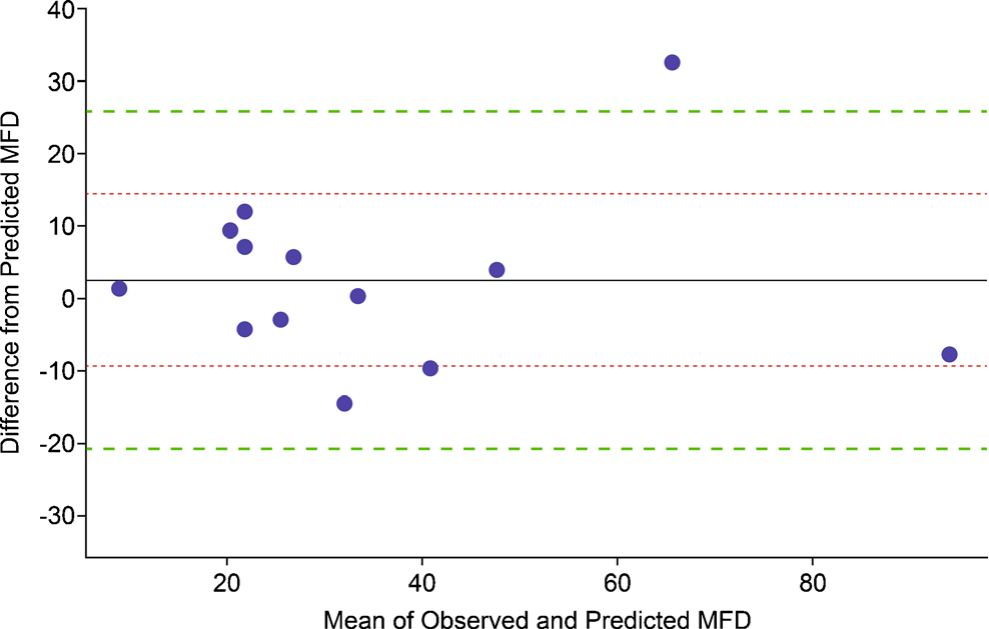
Bland-Altman Plot – the x –axis represents means (i.e., half the sum of observed and predicted MFD values); the y – axis represents the differences (i.e., predicted MFD values subtracted form observed values). The *solid horizontal line* is the mean difference (2.6 NGFs) for our data (*n* = 13) which have coefficient of determination r^2^ = 0.003 (95 % CI 0.000–0.038), showing no trend between means and differences. The *inner dotted horizontal lines* are at one standard deviation (11.9 NGFs) from the mean difference; the *outer dotted lines* are at 1.96 standard deviations (23.3 NGFs). About 68 % of observations will have a difference in MFD between the inner limits of agreement; about 95 % of observations will have a difference in MFD between the outer limits of agreement

Visual comparison of the observed and expected MFDs for the age range 16–37 years shows good agreement (Fig. 2), as does the plot of the observed and predicted MFD values compared to the line denoting an exact match between observed and predicted MFD (Fig. 3). The Pearson product-moment correlation coefficient is 0.87 (95 % CI 0.62–0.96, *p* = 0.001). Bland-Altman analysis shows no proportional error when using predicted MFD values to estimate observed MFD values, since the coefficient of determination is close to zero, with r^2^ = 0.003 (95 % CI 0.000–0.038) for means of observed and predicted values against differences between observed and predicted values (Fig. 4). The mean difference between observed and predicted MFD values is 2.6 NGFs, with 68 % of new values expected to lie within one standard deviation (i.e., 11.9 NGFs) of this mean, and 95 % of new values expected to lie within 1.96 standard deviations (i.e., 23.3 NGFs) (Fig. 4, dashed lines).

This combination of high correlation, low mean difference, tight limits of agreement and lack of proportional error satisfies the criteria for external validation of the predictive model by the observations.

### Published data

Identical analyses were performed using 15 age-related MFD values [25]. Two of the four criteria for external validation were satisfied: the Pearson product-moment correlation coefficient for these data is 0.90 (95 % CI 0.73–0.97, *p* < 0.001), and the mean difference between observed and predicted MFD values is 3.3 NGFs.

Proportional error is small - r^2^ = 0.012 (95 % CI 0.0003–0.273) - but is slightly over the criterion value of 10 %. Limits of agreement at one standard deviation are 20.8 NGFs, which is outside the criterion cut-off of 15 NGFs.

We conclude that these data come close to externally validating the predictive model, failing only on marginally higher proportional error and Bland-Altman limits of agreement. The criteria and results for both sets of observed data are shown in Table 3.

## Discussion

We show a novel predictive model for MFD in the human ovary, and that this has high correlation with our own observed MFD calculations, low mean difference, tight limits of agreement and lack of proportional error. The model can therefore be used to accurately and reliably predict the actual MFD in the cortex of healthy women aged 16 through 37 years (Table 2). The obvious advantage of using the model is that it is non-destructive: no cortical tissue is needed to make the prediction.

Our model depends on one assumption and a calculated estimate of a key parameter. The assumption is that larger ovaries contain more NGFs than small ones, with the proportion roughly linear with ovarian volume. Indirect supporting evidence for this assumption is provided by studies that show strong correlation between ovarian volume and NGF populations [28, 29]. The key parameter is our estimate of the volume of the cortex as a proportion of the entire ovary, which we take to be close to the typical case; it should be noted that these data derive from normal healthy women, in the absence of factors known to affect the key variables such as the use of hormonal contraception which has been shown to significantly reduce ovarian volume [30]. Our estimate of roughly 24 % of ovarian volume can be compared to descriptions of average widths of the cortex seen in histological samples. Our calculations suggest that the width of the cortex is typically between 0.75 and 1.75 mm. There is a lack of homogeneity in the literature regarding these values. Our lower bound is in good agreement with the 0.75 mm quoted in a recent review by Silber [7], and broadly concordant with the 1 to 2 mm range described by von Woolf et el. [8], but our entire range is below the 2–3 mm described by Barrett and Woodruff [31]. We believe that it is reasonable to assume that the volume of the cortex relative to the whole ovary changes little during the age range for this study (i.e., mid teen to late thirties) as there are no published reports of cortical loss due to thinning before menopause [32] however this assumption cannot be extrapolated to earlier ages, since the cortex volume corresponds to about 90 % of the fetal and 85 % of the infant ovary [33]. Moreover cortical follicle density has been reported as up to eight-fold greater in infant and very young ovaries compared to mid teenage tissue [23] highlighting the difference in follicle population at pre-reproductive age.

We combine two predictive models to predict MFDs. The normative model for ovarian volume [20] was validated during development, but the NGF population model [1] has not yet been directly validated. Indirect validation for the model (and hence its suitability for use in this study) follows from its ability to predict average age (and ranges) at menopause, and its close agreement at fertile ages with indirect markers of ovarian reserve such as AMH [2] and ovarian volume [28].

Our own data validated the model, achieving or exceeding the four criteria set out in advance. However we found only partial external validation when data from a published study, employing less stringent counting methods, was used. Two of the four validation criteria were satisfied, with the remaining two being close to the a priori cut-offs (Table 3). A plausible explanation for results that are similar but not quite as close-fitting as for our own data, is that the published MFDs were obtained by examination of half the tissue with correction factors applied, rather than examination of all the tissue. In all studies using differential counts of follicles there is a compromise reached between data accuracy and effort required to generate it and so most studies have opted for a sampling frequency. In this study every section was counted and repeat counts show a high degree of reproducibility with a low coefficient of variation. Hence the MFD calculations for our data are precise (modulo observer error), whereas the published data are reasonable approximations to the actual MFDs. Combining the internal and external data gives 28 observations for the age range 16–37 years. This relatively small sample size for a 21-year age range is a limitation of our study, however, a strength of this study is that we have counted and classified follicles in every section of all tissue samples. There is variation in follicle density and if a sampling frequency is used (e.g. every 10th or 20th section) then results will be skewed. High-quality estimates of MFD depend on access to tissue and intensive laboratory examination, and are therefore hard to obtain. Despite this, it should be possible to revise and improve our predictive model if and when new data become available.

In conclusion, we report development of a model of non-growing follicle density in the human ovarian cortex, which shows excellent fit with newly-derived experimental data. This model will be valuable for assessment of MFD in ovarian biopsies in a range of pathological and experimental situations, including assessment of gonadotoxicity induced by chemotherapy, the development of approaches to mitigate such damage, and potential effects of environmental exposures where only post-treatment/exposure sampling is possible.
